# A Novel Method to Image Macropinocytosis *in Vivo*

**DOI:** 10.3389/fnins.2018.00324

**Published:** 2018-05-15

**Authors:** Lunhao Chen, Daxiao Cheng, Jiachen Chu, Ting Zhang, Zhuoer Dong, Huifang Lou, Liya Zhu, Yijun Liu

**Affiliations:** ^1^Department of Neurobiology, Key Laboratory of Medical Neurobiology, Ministry of Health of China, Zhejiang Provincial Key Laboratory of Neurobiology, Zhejiang University School of Medicine, Hangzhou, China; ^2^Department of Orthopedic Surgery, The First Affiliated Hospital, Zhejiang University School of Medicine, Hangzhou, China; ^3^Department of Physiology, Johns Hopkins University School of Medicine, Baltimore, MD, United States; ^4^Middle School Attached to Northwestern Polytechnical University, Xi'an, China

**Keywords:** macropinocytosis, live imaging, *Drosophila*, embryo, hemocyte, *in vivo*

## Abstract

Here we described an experimental protocol for *in vivo* imaging of macropinocytosis and subsequent intracellular events. By microinjection, we delivered fluorescence dextrans together with or without ATPγS into transparent *Drosophila* melanogaster embryos. Using a confocal microscope for live imaging, we monitored the generation of dextran-positive macropinosomes and subsequent intracellular events. Our protocol provides a continent and reliable way for investigating macropinocytosis and its underlying mechanisms, especially when combined with genetic strategies.

## Introduction

In eukaryotic cells, macropinocytosis is the most efficient way to internalize extracellular fluid through plasma membrane-formed large vacuoles called macropinosomes (Racoosin and Swanson, 1993; Swanson and Watts, 1995; Lim and Gleeson, 2011). As an ancient cellular behavior, macropinocytosis is essential for many physiological and pathological processes, such as nutrients uptake, pathogen capture, antigen presentation, and tumorigenesis (Kerr and Teasdale, 2009; Diken et al., 2011; Liu and Roche, 2015; Bloomfield and Kay, 2016). Sharing similar intracellular mechanism, macropinocytosis is thought to be largely homologous to phagocytosis, neuronal bulk endocytosis and other actin-driven endocytosis (Bloomfield and Kay, 2016).

Macropinocytosis provides a non-selective route to internalize extracellular fluids. In cancer cells, macropinocytosis is utilized for nutrient uptake to support metabolic needs and promote tumor growth (Commisso et al., 2013). Several infectious pathogens, such as bacteria, virus and protozoa, opportunistically hijack macropinocytosis to invade host cells and evade immune responses (Haraga et al., 2008; Gobeil et al., 2013). Observation of macropinocytosis will provide insight into the underlying regulatory molecular mechanisms and enable the physiological control of macropinocytosis for drug delivery in anti-cancer or -infection therapies. However, most observations of macropinocytosis were obtained from *in vitro* experiments or unicellular organisms (Chubb et al., 2000; Chen et al., 2015), e.g., *Dictyostelium* amoebae, instead of naturalistic models that do not fully reflect the complexity of *in vivo* situations, limiting their application. Therefore, considerable gaps remain in the knowledge of the relevance of macropinocytosis, especially the lack of optical imaging approaches, in living organisms. It is essential to develop consistent and reliable methods for *in vivo* macropinocytosis studies.

Most *Drosophila* melanogaster (fruit fly) genes are evolutionarily conserved with human and other mammals (Reiter and Bier, 2002). With its short life cycle and genetic amenability, *Drosophila* provides attractive model systems for various researches (Brand and Perrimon, 1993). After removal of chorions, *Drosophila* embryos become transparent, but still tolerance to subsequent operations for live imaging, rendering this model feasible for *in vivo* cell behavioral and cell biological studies.

In the present study, we described a protocol for *in vivo* studies of macropinocytosis. By microinjection, fluorescence-labeled dextrans were delivered into *Drosophila* embryos. Engulfed by *Drosophila* hemocytes, which resemble mammalian macrophages, fluorescent dextrans were internalized with associated membrane, resulting in formation and subsequent transportation of macropinosomes. For microscopic methods, macropinocytosis was fluorescently visualized and monitored in live embryos. This method provides a novel way for observation of the organization and subsequent processing of macropinosomes *in vivo*, and an ideal model for revealing the underlying mechanisms of macropinocytosis.

## Materials and methods

### Drosophila stocks

A stable line *srp-Gal4;UAS-GFP* was used to visualize hemocytes with green fluorescent protein (GFP) in embryos. F1 embryos were crossed from *Srp-Gal4* and *UAS-tau-GFP* for microtubule labeling in the hemocytes. All crosses were raised on standard *Drosophila* medium at 25°C with 12:12 h light/dark cycle. The *Drosophila* line *srp-gal4; UAS-GFP line* is a kindly gift from Prof. Henry Sun.

### Regents and equipment

#### Injection solutions

Hank's Balanced Salt Solution (HBSS, Invitrogen, Carlsbad, CA, USA) was used for dilution of fluorescent dextrans and adenosine 5 = -O-(3-thio) triphosphate (ATPγS, Sigma-Aldrich, St. Louis, MO, USA) to the final concentration of 5 mg/ml and 1 mM, respectively. Cascade blue labeled 3-kDa fluorescent dextran (CB3S), tetramethylrhodamine (TRITC) labeled 3-, 10-, 40-, and 70-kDa fluorescent dextrans (TRD3S, TRD10S, TRD40S, and TRD70S) were all purchased from Invitrogen.

#### Heptane glue

To stick and stabilize embryos for subsequent injection, heptane glue was prepared as previously described (Brust-Mascher and Scholey, 2009). In brief, one pack of double sticky tapes were unrolled and dissolved with 50 ml heptane (Ourche, China). Seal the bottle and mild shake the solution for at least 12 h until it is clear and sticky.

#### Juice agar plates

Combine 2 g agar with 100 ml fruit juice, add ddH_2_O to a final volume of 250 ml. Boil in microwave and pour the solution into 60 mm diameter Petri dishes and cool down at room temperature for 1 h. Scatter some dry yeast on the surface before use.

#### Micropipettes

Micropipettes for microinjection of dextrans are pre-pulled from borosilicate glass tubes (outer diameter: 1.0 mm, inner diameter: 0.5 mm, BF100-50-15, Sutter Instrument, Novato, CA, USA) by a micropipette puller (P-97, Sutter Instrument) to form a tip of ~5 μm in diameter. A microforge (MF-830, Narishige, Japan) was used for quality control.

#### Glass coverslips

24^*^24 mm and 55^*^24 mm coverslips with thickness 0.13–0.16 mm were used (Stars, China).

### Microinjection preparation

The microinjection system was adapted from micropipette assay system for microglia migration as described previously (Wu et al., 2014). In short, the nitrogen cylinder was connected with Picospritzer (Picospritzer III, Parker Hannifin, Cleveland, OH, USA) and set the output pressure to 0.1 MPa. Connect the “OUTPUT” signal of the electronic stimulator with the micropipette holder and attach the holder to the micromanipulator (MP-225, Sutter Instrument). Set the pulse “DURATION” at 50–100 ms. About 1-2 μl injection solution with or without ATPγS was filled into the micropipette by a 1 ml syringe and make sure without any air bubbles. By local micropipette ejection (Lohof et al., 1992), injection solution is pulse-ejected into embryos controlled by pressing the “MANUAL” button.

### Embryos preparation (Figure 1A)

#### Step 1. embryos collection

100-200 adult *Drosophila* were transferred into an embryo collection cage (Brust-Mascher and Scholey, 2009) and adapt for 1 day before collection. When collection starts, change juice plates and collect newborn embryos per hr. Collected embryos were incubated for at least 5 h until hemocytes matured and GFP expression (Tepass et al., 1994; Miller et al., 2002).

**Figure 1:**
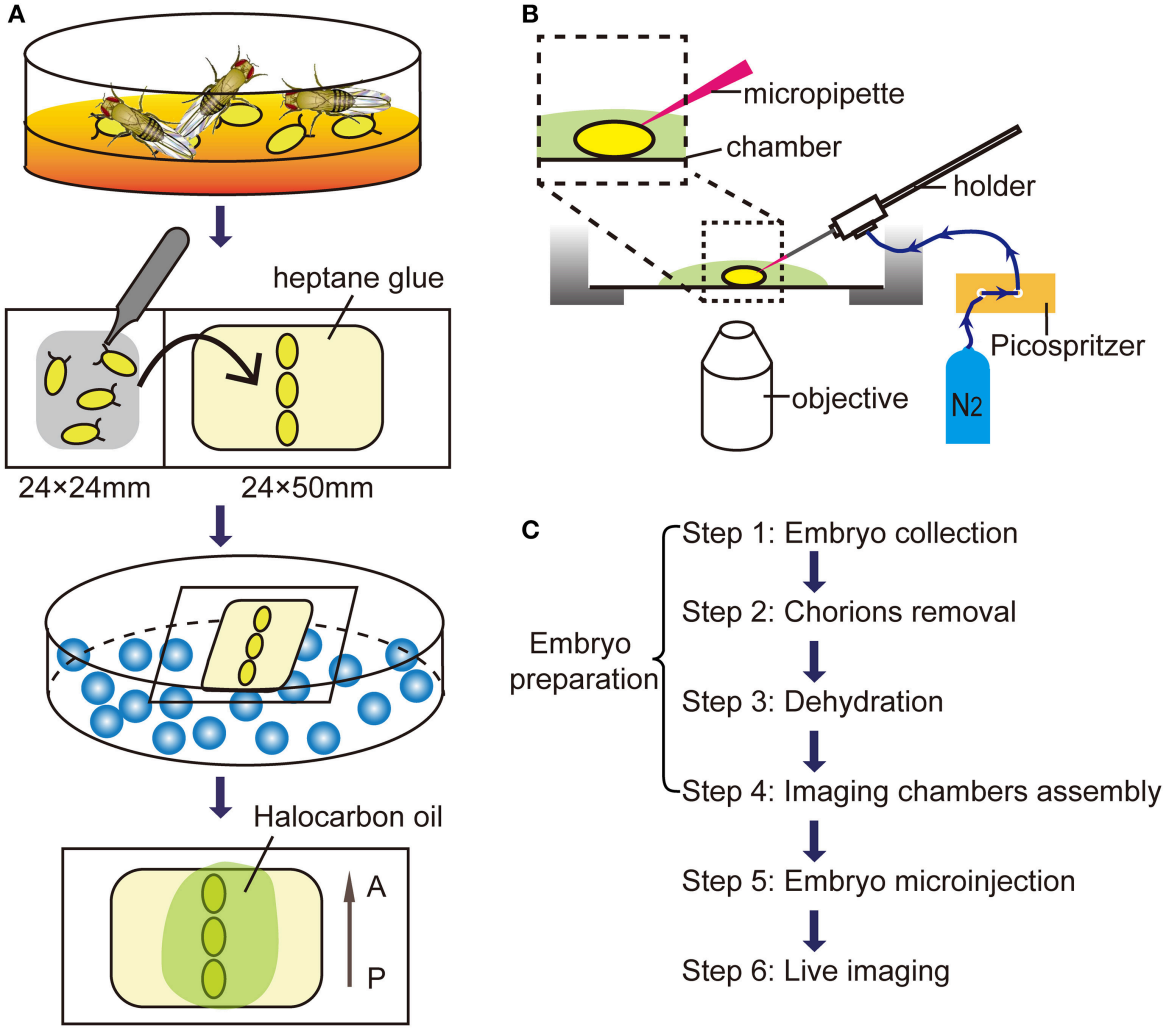
Microinjection procedures for *in vivo* imaging. **(A)** The schematic diagram of embryos preparation. A, anterior; P, posterior. **(B)** The diagram of the microinjection system. **(C)** The flow diagram for live imaging of macropinocytosis in embryonic hemocytes.

#### Step 2. chorions removal

Under a stereoscopic microscope (SZ51, Olympus, Japan), chorions were carefully removed from embryos by rolling them on the double sticky tape by fine tweezers (0208-5-PO, Dumont, Swiss). After removal of chorions, transparent embryos without chorions were paralleled arranged and attached on a coverslip with heptane glue.

#### Step 3. dehydration

Put embryos with the coverslip into a dryer which bottom filled with allochroic silicates (Sinopharm, China). Embryos were dehydrated for ~5 min to prevent leakage of body fluids.

#### Step 4. imaging chambers assembly

Transfer dehydrated embryos to an imaging chamber with a cover glass bottom. A droplet (about 20 μl) of Halocarbon oil 700 (H8898, Sigma-Aldrich) was added to each embryo providing appropriate humidity and enough oxygen.

### Embryo microinjection

Assembly the injection equipment and imaging chambers as shown in the Figure 1B. Under a confocal microscope (FV1200, Olympus) with a 60x/NA 1.2 water objective, move the micropipette tip to the abdominal level of embryos by the micromanipulator. Carefully move the embryo against the micropipette tip and make sure the tip sticking into the embryo at the center of the optic field. Wait for 30 sec and press the “MANUAL” button to operate a single pulse of 10-ms duration. The total injection volume is about 20–30 nl.

### Live imaging

After the dextran injection, 3D Time-lapse imaging was captured using 60x water dipping objectives, stacks of images were acquired with a step size of 1 μm for a depth of 10 μm below embryo surface. Time-lapse movies were then generated between 3D stacks for 90 min without an interval. The time-lapse stack images were reconstructed and analyzed using Imaris software (Bitplane AG) and Image J (National Institute of Health, USA) software. The flow chart from embryo collection to live imaging was illustrated in Figure 1C.

### Statistical analysis

Statistical analysis was performed with STATA software (Version 13.0, Stata Corp, USA). Data are presented as means ± standard errors of the means (SEM). Statistical comparisons were assessed using student *t*-test or one-way ANOVA among three groups or above. Differences were considered to be significant at a P level of <0.05.

### Troubleshooting

Troubleshooting advices can be found in Table 1.

**Table 1:** Troubleshooting table.

**Problem**	**Possible reason**	**Solution**
Embryos are carried away during inserting.	Embryos are glued not enough.	Prepare thicker heptane glue to stable embryos.
	The tip is not sharp enough.	Adjust the tip size of micropipettes during preparation.
Body fluids flow out during injection.	Not enough Dehydration.	Prolong dehydration to 6-7 min.
	The flow from the micropipette tip is too high.	Use the micropipette with an appropriate tip size.
		Reduce the output pressure or the pulse duration.
Dextran-positive macropinosomes cannot be seen after 30 min of injection.	The micropipette tip is sealed or its tip is too small.	Replace the micropipette with a newly-made one.
		Use the micropipette with an appropriate tip size.
	Air bubbles are trapped in the micropipette tip.	Gently flick the micropipette to discharge bubbles or reload the injection solution.
	Large dextrans are filtered during diffusion.	Use small dextrans. 3 kDa-dextran is recommended.
	Embryos are not healthy enough.	Use another healthy embryo.
		Operate embryos as quickly as possible.
High background in the extracellular space	The micropipette tip is too large.	Adjust the tip size of micropipettes.
	The output pressure is too high or pulse duration is too long.	Reduce the output pressure or the pulse duration.

## Results

Using our embryo microinjection method, we first sought to determine whether embryonic hemocytes were capable for evoking macropinocytosis. After induction by ATPγS, a non-hydrolysable ATP analog, macropinocytosis was observed using a confocal microscope. Five standard rules are followed to identify formed macropinosomes; (1) dextran-positive; (2) approximately pellet and ellipse-shaped; (3) surrounded by GFP-positive cytoplasm;(4) larger than 0.2 μm in diameter; (5) fluorescence intensities of macropinosomes are comparable with or higher than that of the extracellular space.

**Figure 2:**
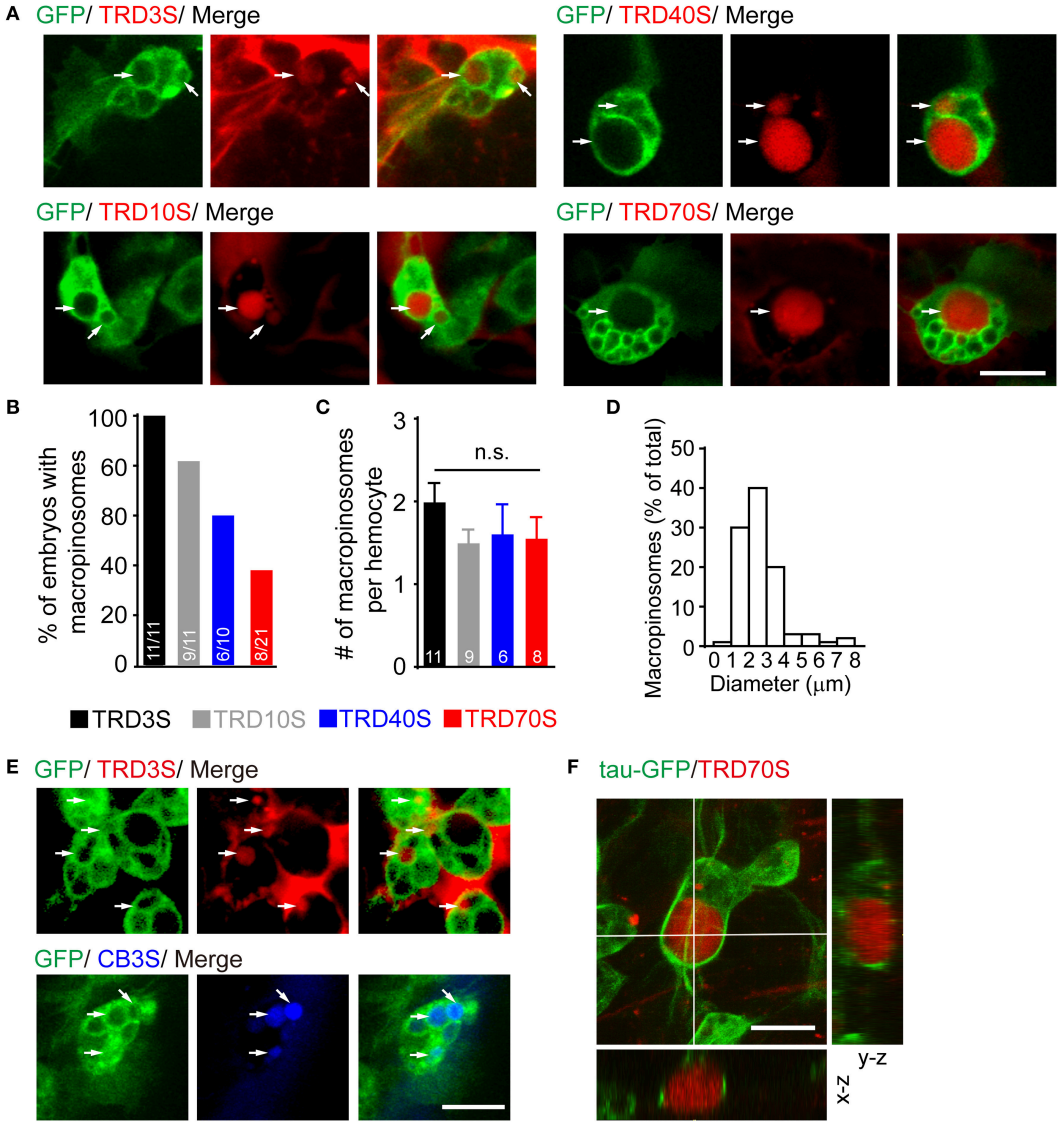
ATPγS induced macropinosomes in *Drosophila* embryonic hemocytes. **(A)** Hemocytes (green) uptook fluorescent dextrans ranged from 3-kDa (TRD3S) to 70-kDa (TRD70S) dextrans and generated macropinosomes (arrows). **(B)** Success rates were defined as the percentage of embryos where hemocytes with macropinosomes 1.5 h after completion of different-sized dextrans injected. **(C)** Numbers (#) of macropinosomes in each hemocyte. *P*-values were calculated using one-way ANOVA among groups; n.s.: non-significant. **(D)** The size-distribution pattern of macropinosomes containing TRD3S (*n* = 100 macropinosomes, from 11 embryos). **(E)** Both TRITC labeled (red, TRD3S) and cascade blue labeled (blue, CB3S) dextrans were feasible to be uptaken by GFP-positive hemocytes (green), and formed macropinosomes (arrows). **(F)** In-depth 3D reconstruction analysis of ATPγS induced macropinosomes using a spatial deconvolution. Note that TRD70S dextran (red) labeled macropinosomes (red) and their surrounding GFP-positive microtubule-structures (green). Images were displayed in x-y (top), x-z (bottom), and y-z (right) projections. Scale bars, 10 μm.

In our observation, responding to injected ATPγS, GFP-positive hemocytes efficiently engulfed large volumes of extracellular fluids containing fluorescent-labeled dextran with diverse molecular weights and different fluorophores (Figures 2A,E). However, macropinosomes were seen in almost 100% of embryos after 3-kDa dextran injection, whereas only 81.8, 60.0, and 38.1% of embryos generated macropinosomes after injection of 10, 40, and 70-kDa dextran, respectively (Figure 2B). To clarify whether hemocytes have equal ability to uptake dextrans with different sizes, we measured the number of macropinosomes in each hemocyte. Our result shows that about 2 macropinosomes were generated in each hemocyte and there were no statistically significant differences among hemocytes uptake 3-kDa to 70-kDa dextrans (Figure 2C). In addition, our result showed that dextran-positive pinosomes were heterogeneous in sizes, ranging from 0.2 to 10 μm, and approximately 90% of the macropinosomes were 1 to 4 μm in diameter (Figure 2D). Combined with genetic strategies, e.g., GAL4/UAS system, this model is appropriate for molecular studies, especially in endocytosis and its subsequent events. For instance, using hemocyte-specific promoter *srp-Gal4* to drive GFP fused tubulin-associated protein tau (tau-GFP), microtubule-filaments could be visualized and further analyzed in formation and trafficking of nascent macropinosomes (Figure 2F).

**Figure 3:**
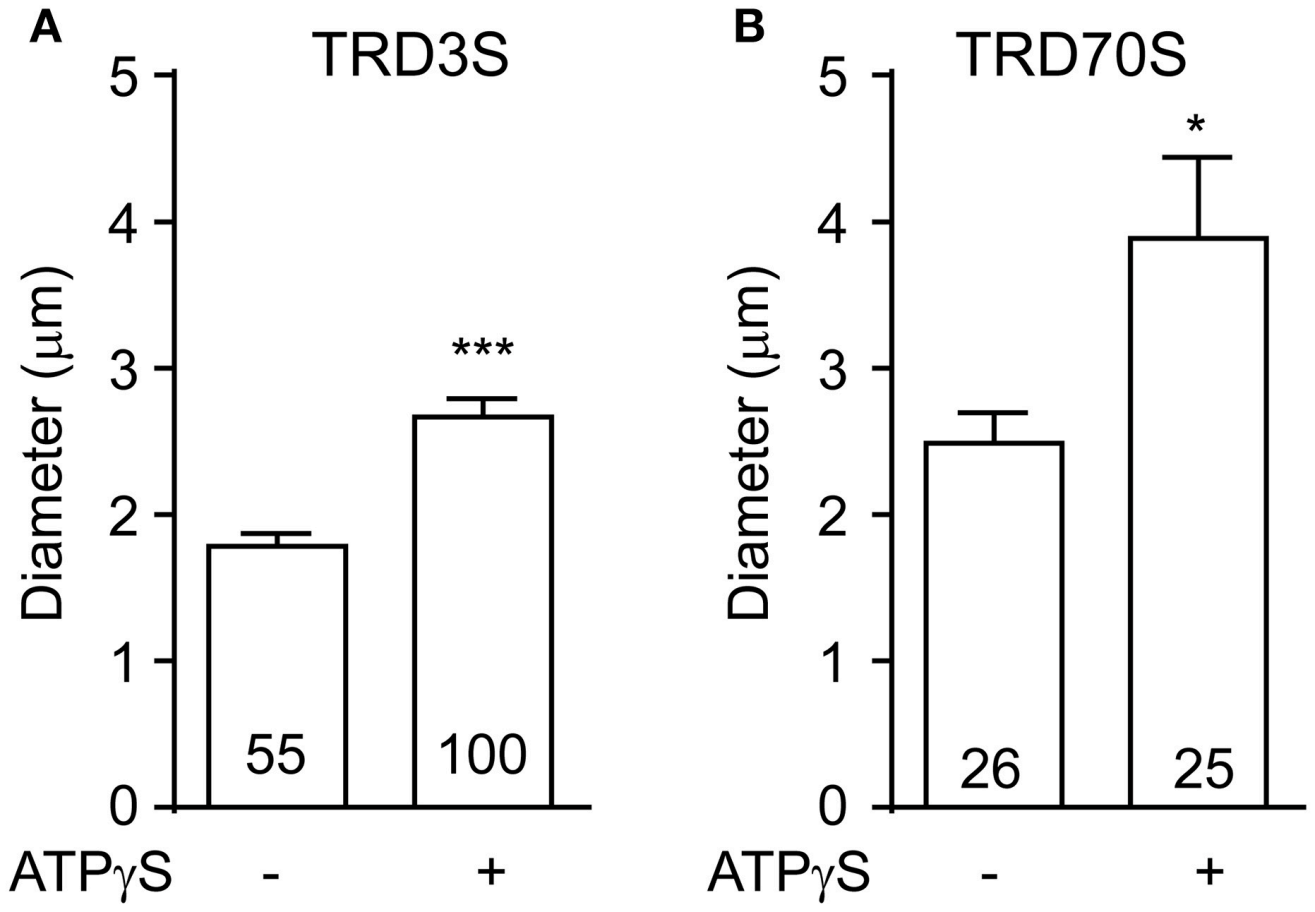
ATPγS stimulation increased the vesicle size of macropinosomes. The diameter of macropinosomes were analyzed at 1.5 h after TRD3S **(A)** or TRD70S **(B)** injected. *P*-values of significance (indicated with asterisks, ^*^p < 0.05, ^***^p < 0.001) were calculated using student *t*-test.

To clarify whether ATPγS was necessary for hemocytes to induce macropinosomes in our system. We injected 3 or 70-kD dextrans without ATPγS into embryonic hemocyte and observed spontaneous macropinocytosis in GFP positive hemocytes. Compared with ATPγS induced macropinosomes, spontaneous macropinosomes were much smaller (Figure 3), raising the difficulty for *in vivo* observation. Therefore, we recommend ATPγS induction to promote macropinocytosis in this model.

**Figure 4:**
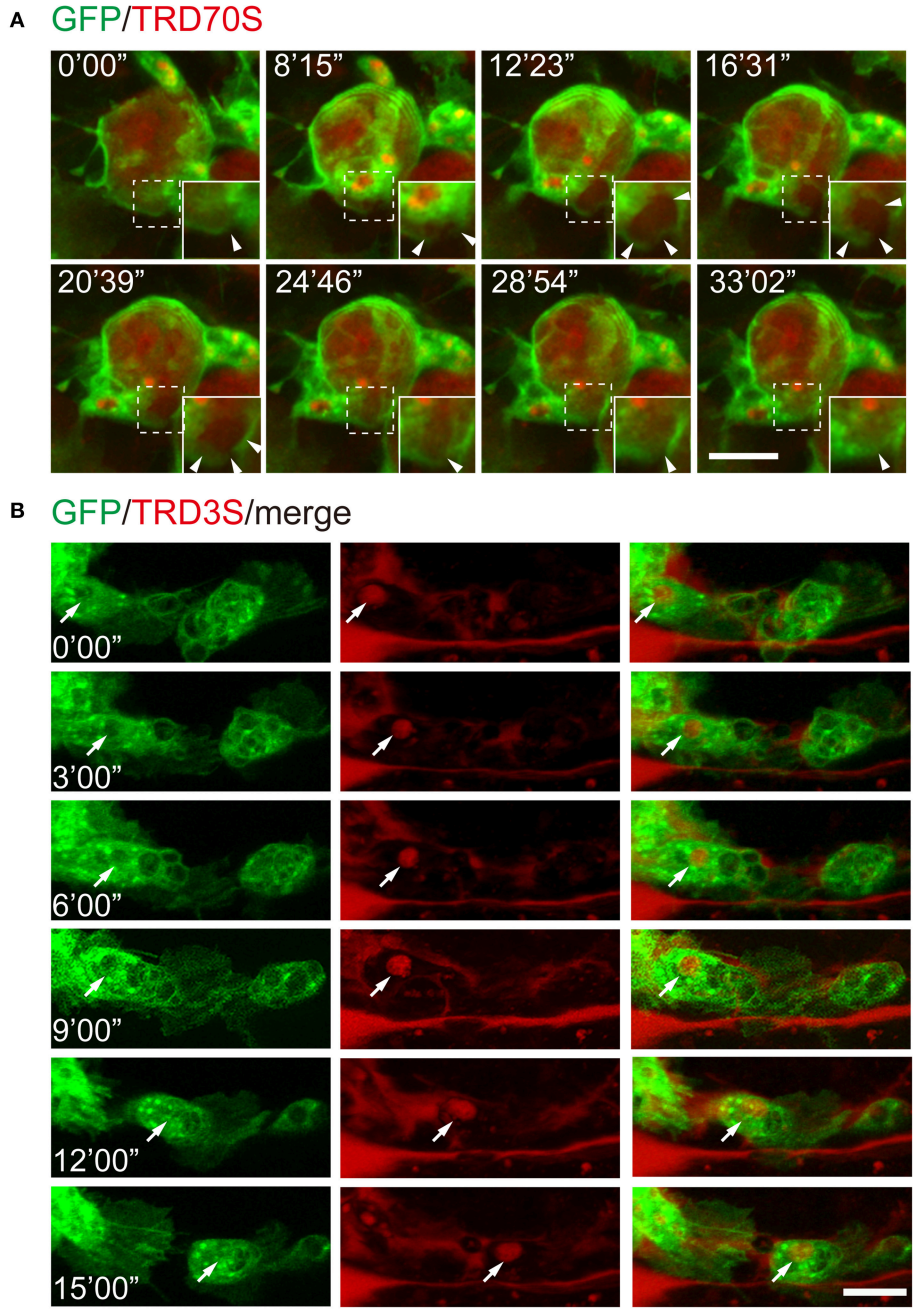
Recording of cellular and subcellular behaviors using the ATPγS microinjection-based imaging system. **(A)** Dynamic changes of macropinosomes, from formation to fusion. Note that macropinosomes (red) were formed from the surface membrane ruffles and internalized after enclosure (arrowheads). The newly formed macropinosomes were indicated by dashed boxes and corresponding enlarged parts were presented at right corners. **(B)** TRD3S macropinosomes (red) transported within migrating hemocytes (green). The arrow indicates the location of a trafficking vesicle. Scale bars, 10 μm.

To further test whether this method is suitable for monitoring the cellular and subcellular events of macropinocytosis, we injected fluorescence-labeled dextrans together with ATPγS into the embryos with GFP-labeled hemocytes. As shown in Figure 4A, GFP-hemocyte extracellular dextrans were internalized along with cell surface ruffling and generated TRD70S-positive macropinosomes. Using low molecular weight TRITC-dextrans, TRD3S, we monitored the intracellular events of ATPγS-induced macropinosomes. During a 15 min observation period, dextran-containing macropinosomes transported in the cell body of a migrating hemocytes, indicating the method is reliable for cellular events and cell behavioral recordings (Figure 4B).

## Discussion

Adenosine triphosphate, or ATP, is the principal molecule for intracellular energy transfer in cells. Extracellular ATP is also an essential messenger for several physiologic and pathological processes (Lim and Gleeson, 2011; Li et al., 2013; Cisneros-Mejorado et al., 2015). Sensed by purinergic receptors, extracellular ATP activates intracellular signaling pathway and induces membrane ruffling and macropinocytosis (Grimmer et al., 2002; Li et al., 2013).

Here, we present an ATP analog microinjection-based system for *in vivo* observation of macropinocytosis and its subsequent cellular events. In our observation, ATPγS are capable to induce larger macropinosomes engulfed by *Drosophila* embryonic hemocytes using different molecular weights of dextrans (Figures 2, 3). In addition, small dextrans are more efficient for labeling macropinosomes. Moreover, this method is feasible to investigate cellular events of macropinosomes, such as generation, fusion, and trafficking (Figure 4).

Although macropinocytosis is a nonselective process in cell culture systems, natural structures in living tissues may filter molecules and cause different diffusion properties in different size dextrans. Therefore, we tried to find out which size of dextran would be suitable for our system. In our experiment, dextrans ranged from 3-kDa to 70-kDa were all capable of labeling macropinosomes. However, small dextran (TRD3S) labeled macropinosomes could be seen in almost 100% of injected embryos, whereas large dextran (TRD70S) could only successfully label macropinosomes in about 40% of embryos under same conditions (Figure 2B). These data suggest that small molecules are more efficient for labeling macropinosomes in our system. In addition, although induced by different-sized dextrans, each hemocyte generated approximate numbers of macropinosomes. Taken together, application of larger dextran will reduce the success rate of induction without changing the uptake ability of hemocytes. It also suggests a potential filter effect of extracellular matrix in live tissues.

There are some difference of macropinocytosis between *in vivo* and *in vitro* systems. In previously studies, cultured macrophages and microglia exhibit impressive capabilities in internalization of extracellular fluids by macropinosomes (Racoosin and Swanson, 1993; Chen et al., 2015; Canton et al., 2016; Fu et al., 2016), which could generate within 1 min followed by centripetally migration and rapid shrink (Racoosin and Swanson, 1993; Lee and Knecht, 2002). It is a relatively short window to observe each phase. By contrast, *in vivo* macropinocytosis takes longer to generate macropinosomes in about 30 min (Figure 3A), providing enough time for observation. In addition, primary macrophages and the Drosophila hemocyte S2 cell line generate macropinosomes with high density (Gupta et al., 2009; Canton et al., 2016), raising the difficulty for observation of each vesicle. In contrast, no more than 5 macropinosomes were observed in our *in vivo* model. The sparse labeling provides a convenient approach to distinguish and monitor macropinosomes.

Combined with genetic tools and other strategies, this method is suitable to investigate molecular functions in macropinocytosis. For instance, using *srp-GAL4;UAS-tau-GFP* transgenic *Drosophila*, microtubules were visualized around ATPγS induced macropinosomes (Figure 2F). Together with previous *in vitro* studies (Gilberti and Knecht, 2015), our results provide essential *in vivo* cues supporting that microtubule-associated structures may regulate macropinosomes formation and subsequent processes.

To expend applications, the method could be used to: (1) reveal the underlying mechanism in macropinocytosis; (2) uncover the way by which macrophages/microglia used for pathogens internalization; (3) develop new approaches for drug delivery via macropinocytosis. Taken together, this method provides novel sights for *in vivo* investigation of macropinocytosis and associated processes.

## Ethics statement

This study was carried out in accordance with the recommendations of the Guidance for the Care and Use of Laboratory Animals at Zhejiang University. The protocol was approved by the Institutional Animal Care and Use Committee at Zhejiang University.

## Author contributions

LC and YL designed the research. LC, DC, JC, TZ, ZD, and YL performed the experiments together; LC, JC, and YL wrote the paper; LZ and HL helped with animal raising and experimental preparation; LC with JC analyzed the data; YL supervised the entire study. All authors discussed the results and commented on the manuscript.

### Conflict of interest statement

The authors declare that the research was conducted in the absence of any commercial or financial relationships that could be construed as a potential conflict of interest.
